# 17β-Estradiol Enhances Schwann Cell Differentiation via the ERβ-ERK1/2 Signaling Pathway and Promotes Remyelination in Injured Sciatic Nerves

**DOI:** 10.3389/fphar.2018.01026

**Published:** 2018-10-09

**Authors:** Yun Gu, Yumen Wu, Wenfeng Su, LingYan Xing, Yuntian Shen, Xiaowen He, Lilan Li, Ying Yuan, Xin Tang, Gang Chen

**Affiliations:** ^1^Key Laboratory of Neuroregeneration of Jiangsu and Ministry of Education, Co-innovation Center of Neuroregeneration, Nantong University, Nantong, China; ^2^Jiangsu Clinical Medicine Center of Tissue Engineering and Nerve Injury Repair, Affiliated Hospital of Nantong University, Nantong, China; ^3^Affiliated Hospital of Nantong University, Nantong, China; ^4^Department of Anesthesiology, Affiliated Hospital of Nantong University, Nantong, China

**Keywords:** 17β-estradiol (E2), myelination, estrogen receptors (ERs), ERK1/2, lysosome, peripheral nerve injury

## Abstract

Remyelination is critical for nerve regeneration. However, the molecular mechanism involved in remyelination is poorly understood. To explore the roles of 17β-estradiol (E2) for myelination in the peripheral nervous system, we used a co-culture model of rat dorsal root ganglion (DRG) explants and Schwann cells (SCs) and a regeneration model of the crushed sciatic nerves in ovariectomized (OVX) and non-ovariectomized (non-OVX) rats for *in vitro* and *in vivo* analysis. E2 promoted myelination by facilitating the differentiation of SCs *in vitro*, which could be inhibited by the estrogen receptors (ER) antagonist ICI182780, ERβ antagonist PHTPP, or ERK1/2 antagonist PD98059. This suggests that E2 accelerates SC differentiation via the ERβ-ERK1/2 signaling. Furthermore, E2 promotes remyelination in crushed sciatic nerves of both OVX and non-OVX rats. Interestingly, E2 also significantly increased the expression of the lysosome membrane proteins LAMP1 and myelin protein P0 in the regenerating nerves. Moreover, P0 has higher degree of colocalization with LAMP1 in the regenerating nerves. Taking together, our results suggest that E2 enhances Schwann cell differentiation and further myelination via the ERβ-ERK1/2 signaling and that E2 increases the expression of myelin proteins and lysosomes in SCs to promotes remyelination in regenerating sciatic nerves.

## Introduction

Axon myelination is essential for rapid salutatory impulse conduction in the vertebrate nervous system during both development and nerve regeneration following injury (Taveggia et al., 2010; Pereira et al., 2012). The multi-layered myelin sheaths around axons are generated by plasma membranes of specialized glial cells: oligodendrocytes (OLs) in the central nervous system (CNS) and Schwann cells (SCs) in the peripheral nervous system (PNS). The malformation or deterioration of the myelin sheath can cause multiple neurological diseases, including multiple sclerosis, Charcot-Marie-Tooth, leukodystrophies, and peripheral neuropathies (Pereira et al., 2012).

17β-estradiol (E2), a nature estrogen in humans and rodents, plays diverse roles in physiological and pathological conditions in the nervous system (Chaovipoch et al., 2006; Acs et al., 2009; Taylor et al., 2010). Recently, studies have suggested that E2 may regulate myelination and remyelination in both the central and peripheral nervous systems (Melcangi et al., 2003; Schumacher et al., 2007; Zhu and Glaser, 2008; Chen et al., 2016). However, the mechanisms by which E2 regulates formation and maintenance of myelin sheath remain poorly understood. Previous reports have shown that the PI3K/AKT/mTOR and ERK/MAPK signaling pathways are required for both OL and SC myelination and further promote myelin repair (Pereira et al., 2009; Gaesser and Fyffe-Maricich, 2016; Gonsalvez et al., 2016). However, whether estrogen promotes peripheral nerve myelination and remyelination via the AKT or ERK signaling pathway remains unknown.

Lysosomes had long been considered as terminal organelles in cell degradation. Interestingly, accumulating studies have shown that lysosomes in specific cells function in secretory process, which can package bioactive substances and release contents via exocytosis (Blott and Griffiths, 2002; Zhang et al., 2007; Ballabio and Gieselmann, 2009). A growing number of studies, including our recently study (Chen et al., 2012; Shen et al., 2016; Su et al., 2016) have shown that the lysosomes of OLs and SCs contain abundant myelin proteins and play an important role in remyelination of injured nerves via the lysosomal exocytosis. In this paper, we would also investigate whether estrogen regulates myelination by enhancing lysosomal exocytosis of Schwann cells.

The goal of this study was to determine the roles of E2 on Schwann cell myelination. We found that E2 promotes SC differentiation and further SC myelination via the ERβ-ERK1/2 signaling pathway *in vitro* and accelerates remyelination of the regenerated axons in crushed sciatic nerves.

## Materials and Methods

### Antibodies, Growth Factors and Chemicals

Growth factors: human heregulin-β1 (HRG) was purchased from Proteintech Group, and 17β-Estradiol (E2), Nerve Growth Factor (NGF), Trypsin, N6, Thy1.1, 5-Fluoro-2′-deoxyuridine (FDU), Uridine (U), Forskolin, L-Ascorbic acid, ITS Liquid Media Supplement, Poly-D-lysine Hydrobromide (PDL), 2′-*O*-Dibutyryladenosine3′,5′-cyclicmonophosphatesodiumsalt (db-cAMP) were from Sigma (St. Louis, MO, United States). Chemicals: MEK 1/2 inhibitor PD98059, phosphoinositide 3-kinase inhibitor LY294002 were from Cell Signaling Technology, E2R inhibitor ICI182780 was from Abcam,andE2Rα inhibitor MPP and E2Rβ inhibitor PHTPP were from Santa Cruz Biotechnology. Antibodies: Chicken anti-myelin protein zero (P0), rabbit anti-phospho-AKT, anti-AKT, anti-phospho-PI3 Kinase, anti-PI3 Kinase, anti-ERK1/2 (ERK), anti-phospho-ERK1/2 were from Abcam, Rabbit anti-myelin associated glycoprotein (MAG), Mouse anti-neurofilament 200 (NF200), anti-β-actin, anti-S100β were from Sigma, and Rabbit anti-ERα and goat anti-ERβ were from Santa Cruz Biotechnology. Other chemicals were obtained from Sigma.

### Cell Culture

All the animal procedures were approved by the Institutional Animal Care & Use Committee (IACUC) of Nantong University. Primary Schwann cells were isolated from sciatic nerves of Sprague-Dawley rats at postnatal day 2 (provided by the Laboratory Animal Center of Nantong University) as previously described (Gu et al., 2014). Dorsal root ganglion (DRG) neurons were prepared from rat embryos at gestational day 14 as previously described with minor modifications (Paivalainen et al., 2008).

### Myelination Assay *in vitro*

An *in vitro* myelination assay was performed by using the well-established rat DRG/SC myelinating coculture model as previously described with minor modifications (Paivalainen et al., 2008; Su et al., 2016). Approximately 50000 Schwann cells were added to each DRG culture in 24-well plastic culture plates with DMEM Medium containing 10% FBS, 50 ng/ml NGF and allowed to attach overnight, and then were cultured in DRG growth medium for 2 days. The DRG/Schwann cell cocultures were further maintained for 4 ∼ 6 days in the differentiation medium [DMEM, ITS (1:100), 0.2%BSA, 50 ng/ml NGF], after which myelination medium (DMEM, 15% FBS, 50 μg/ml ascorbic acid, 50 ng/ml NGF) was added for myelination induction. Myelination was achieved in the following 12 ∼ 28 days, with the culture medium changed every 2 ∼ 3 days.

### Cell Migration Assay

To mimic physiological conditions, fasciculated DRG axons and reaggregated Schwann cells were used for cell migration assay (Yamauchi et al., 2004). DRG neurons were plated onto a PDL-coated dish, in which axons extended and became fasciculated after 2–3 weeks. Schwann cell reaggregation were achieved by plating Schwann cells on a non-permissive substrate overnight with gentle agitation every 2 ∼ 3 h. The reaggregated Schwann cells were then plated onto the fasciculated axons or PDL-coated cover slips. Individual Schwann cell was allowed to migrate out of the reaggregates along the axons of DRGs in the presence of E2 (50 nM) or HRG (10 ng/ml) for 0.5–24 h. Images were collected with a microscope (Leica Imaging Systems, Cambridge, England). The distance of migration was calculated by measuring the size of the reaggregates over time, subtracting the average initial size of the reaggregates, and dividing the remaining distance in half. Experiments were performed in quadruplicate, and data are presented as means ± SEM. Student’s *t*-test was carried out for comparisons between two groups.

### Cell Proliferation Assay

Schwann cells were plated onto the fasciculated axons or PDL-coated cover slips (40000 cells/well) in DMEM containing 10% FBS. One day later, the medium was changed to DMEM containing 1% FBS (non-proliferating medium) for another 1 day, and the cells were subjected to experimentation in the presence of E2 (50 nM) or vehicle (control). The proliferation of Schwann cells was determined by 5-ethynyl-20-deoxyuridine (EdU) labeling according to the manufacture’s instruction (EdU labeling/detection kit, Ribobio, Guangzhou, China). Briefly, 50 μM EdU was added to the cell medium. Twenty-four hours later, the EdU-containing medium was washed out, and cells were fixed with freshly prepared 4% formaldehyde. Afterward, EdU labeling was conducted according to the manufacturer’s instructions. Labeling with Hoechst 33342 (1 μg/ml, Sigma) was also performed. The images were captured on a DMR fluorescent microscope (Leica Microsystems, Wetzlar, Germany), and the percentage of EdU-positive cells was calculated from 10 random fields in 3 wells.

### Cell Differentiation Assay

The Schwann cell differentiation was assessed as described previously with minor modifications (Monje et al., 2009; Arthur-Farraj et al., 2011). In brief, Schwann cells were plated onto the PDL-coated cover slips (40000 cells/well) in DMEM containing 10% FBS for 24 h and were then induced to acquire a differentiated phenotype by exposure for 3 days to 1 mM db-cAMP and 20 ng/ml HRG which were used at stimulation experiments in non-proliferating medium (DMEM/F12, 1% FBS). Western blotting was used to analyze the expression of protein markers for myelinating/non-myelinating SCs, and immunocytochemistry was used to analyze cell morphology.

### Surgical Procedure

Adult Female SD rats, weighing 200–250 g, were provided by the Experimental Animal Center of Nantong University. The experimental procedures were performed according to the institutional guidelines of animal care of Nantong University, and approved by the Administration Committee of Experimental Animals, Jiangsu Province, China. The rats were anesthetized with an intraperitoneal injection of 3% sodium pentobarbital solution (30 μg/g body weight) prior to surgery. To reduce blood estrogen, bilateral ovariectomy (OVX) was performed as described previously (Islamov et al., 2003). In sham mice, ovaries were exposed, and then incision were sutured. Animals were allowed to recover for 3 weeks before the right sciatic nerves were crushed at the level of sciatic notch for 15 s with a fine hemostat. Following the sciatic nerve crush surgery, E2 (0.2 μg/g) or the same volume of saline was injected subcutaneously every day in OVX and sham groups.

### Enzyme Linked Immunosorbent Assay (ELISA)

Whole blood samples were obtained by cardiac puncture at 24 h after E2 or saline injected. Serum was isolated by centrifugation at 3000 × *g* for 5 min and stored at -80°C for further analysis. E2 was measured by enzyme-linked immunosorbent assay (ELISA) kit (R&D Systems, Minneapolis, MN, United States) according to the manufacturer’s instructions.

### Immunocyto/Histochemistry

The cells were fixed with 4% formaldehyde and processed for immunocytochemistry as described previously (Gu et al., 2010). In brief, the cells were blocked in 5% normal serum plus 0.1% Triton X-100 in PBS and incubated with primary antibodies (appropriate dilution) overnight at 4°C, which was followed by incubation with Fluorescein-isothiocyanate (FITC) or tetraethyl rhodamine isothiocyanate (TRITC)-conjugated secondary antibodies (1:400) at room temperature for 1 h. The cells were counterstained with Hoechst 33342 prior to visualization under a fluorescent microscope. Photographs were taken with a camera and image acquisition software.

The sciatic nerves were fixed in 4% formaldehyde and embedded in OTC compound (Miles Scientific) for immunohistochemistry. Longitudinal frozen sections were incubated with primary antibodies (appropriate dilution) before anti-rabbit IgG-Alex-488 conjugated anti-rabbit IgG andCy3 conjugated anti-mouse IgG (1:200) was added as secondary antibodies.

The above-mentioned primary antibodies included rabbit anti-MAG (1:200), rabbit anti-S100β (1:500), mouse anti-Neurofilament 200 (1:200), chicken anti-P0 (1:200), rabbit anti-cathepsin D (1:500), rabbit anti-lamp1 (1:500).

### Western Blotting

In brief, the protein samples were separated by sodium dodecyl sulfate-polyacrylamide gel electrophoresis (SDS-PAGE) and further transferred onto PVDF membranes (Millipore Corp.). After blocking in Tris-buffered saline (TBS, pH 7.6) containing 5% non-fat milk and 0.1% Tween-20, the membranes were probed with primary antibodies at 4°C overnight. After washed with TBST (TBS and 0.1% Tween-20), the membranes were incubated with a horseradish peroxidase (HRP)-conjugated secondary antibodies (Bio-Rad). Proteins were further detected by the SuperSignal West Pico Chemiluminescent Substrate kit (Pierce). The image was scanned with GS800 Densitometer Scanner (Bio-Rad), and processed with PD Quest 7.2.0 software (Bio-Rad).

The above-mentioned primary antibodies included rabbit anti-MAG (1:800), chickenanti-P0 (1:800), mouse anti-β-actin (1:4000), rabbit anti-p-AKT (1:1000), mouse anti-AKT (1:1000), rabbit anti-ERK1/2 (1:1000), rabbit anti-p-ERK1/2 (1:1000).

### Statistical Analysis

Data are presented as means ± SEM. Differences between groups were compared using Student’s *t*-test or one-way ANOVA or two-way ANOVA, followed by Scheffe’s *post hoc* test. The criterion for statistical significance was *p*< 0.05.

## Results

### E2 Promotes Myelination *in vitro*

**FIGURE 1:**
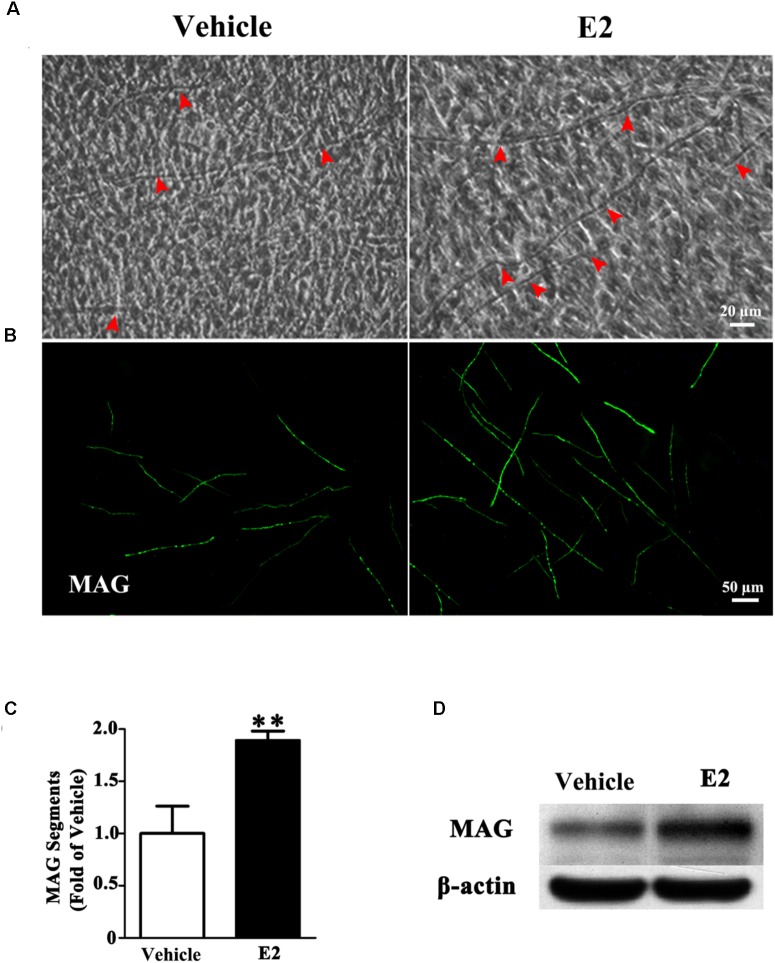
E2 treatment accelerated myelin formation in DRG neurons co-culture with Schwann cells. **(A–D)** Schwann cell/DRG neuron cocultures were grown in myelin-promoting media with or without E2 and maintained for 28 days, the cocultures were photographed **(A)** or fixed and stained for MAG **(B)**, red arrow showing the myelinated axons, scale bar, 50 μm. **(C)** The numbers of myelin sheaths per high-powered field were counted. The ratio of mean values for each E2 and vehicle from three representative experiments are shown. The number of myelin sheaths in the cultures treated with E2 was significantly higher than that in control cultures. ^∗∗^*p* < 0.01, Student’s *t*-test, *n* = 3 cultures per group. All the data are mean ± SEM. **(D)** Western blot for the expressions of myelin proteins MAG, β-actin served as a protein loading control. The results showed the expression of MAG in cocultures with E2 treatment was higher than that without E2.

To investigate whether E2 promotes myelination of Schwann cells *in vitro*, the DRG/SC myelinating co-culture model was used in this study. After 4 weeks of DRG/SC co-culture, a typical myelin-like segregation structures can be observed under phase-contrast microscope (**Figure 1A**), which was confirmed by the expression of the myelin protein MAG with immunohistochemistry (**Figure 1B**). To quantify the extent of myelination, we measured the number of MAG positive myelinated segments. The number of myelin sheath was increased by approximately twofold in the presence of E2 (50 nM) (**Figure 1C**, Vehicle group: 1 ± 0.24; E2 group: 1.89 ± 0.08, *p* = 0.0036, Student’s *t*-test.). Consistent with this, the expression of MAG protein was significantly increased when cells were treated with E2, as shown by western blotting (**Figure 1D**). Together, these results indicated that E2 enhances myelination *in vitro*.

### E2 Enhances Schwann Cell Differentiation Not Proliferation or Migration to Promote Myelination

**FIGURE 2:**
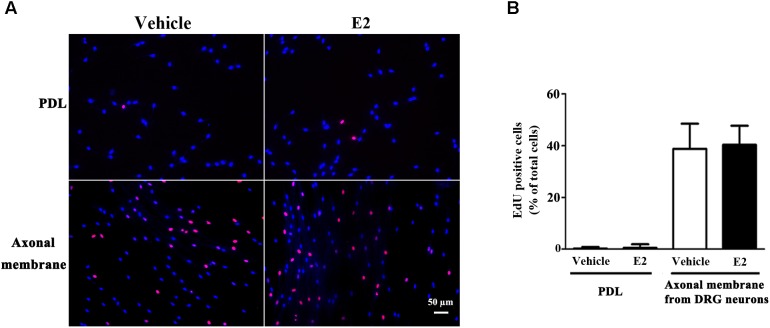
Schwann cell proliferation was not modulated by E2. Schwann cells cultured on fasciculated DRG axons (axonal membrane) or PDL with or without E2 for 24 h, and the cell proliferation was measured by using EdU labeling. **(A)** The images of EdU staining (red) and Hoechst 33342 staining (blue) were used to measure cell proliferation, scale bar, 50 μm. Cell counting was performed in at least 10 random microscopic fields (200× magnification). **(B)** Histograms showing that there were no significant changes in cell proliferation of SCs between E2 treatment or not in cultured on fasciculated DRG axons or PDL. *P* > 0.05, Student’s *t*-test, *n* = 3 cultures per group. All the data are mean ± SEM.

**FIGURE 3:**
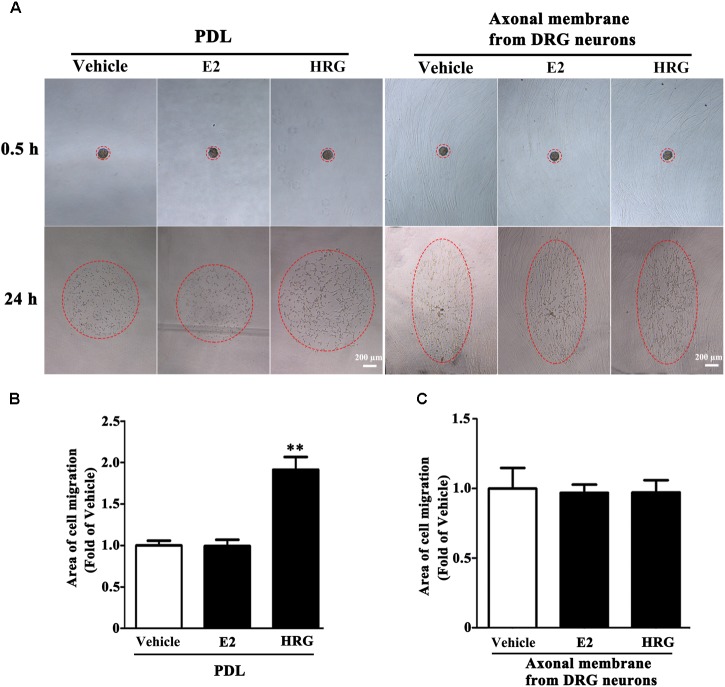
Schwann cell migration was not modulated by E2. Schwann cell reaggregates on fasciculated DRG axons or PDL were incubated with E2 or HRG (Served as positive control), and the area of migration **(A)** was measured, red dashed circle showed the distance of SC migration, scale bar, 200 μm. **(B,C)** Histograms showing that E2 could not stimulate SC migration on both fasciculated DRG axons and PDL, by contrast, HRG significantly increased SC migration from the reaggregates after stimulation with HRG for 24 h on PDL **(B)** but not on DRG axons **(C)**. ^∗∗^*p* < 0.01 versus vehicle group. One-way ANOVA, *n* = 3 cultures per group. All the data are mean ± SEM.

**FIGURE 4:**
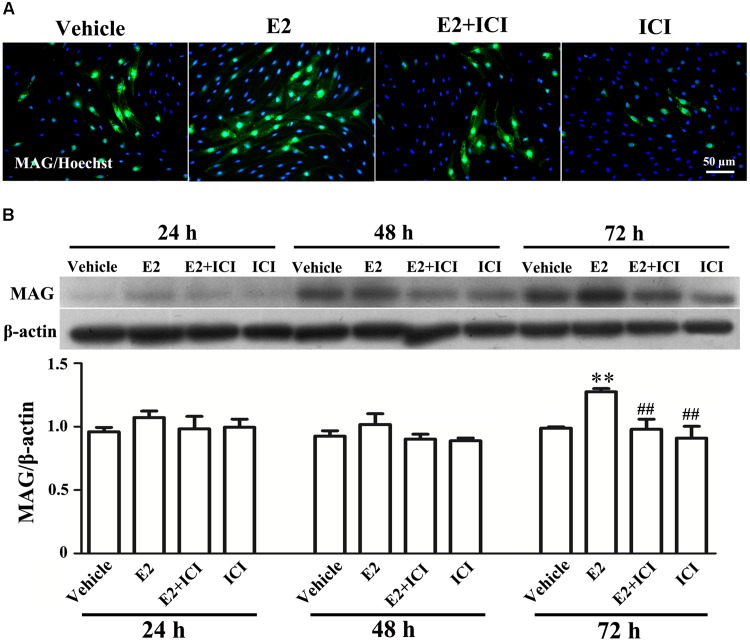
E2 treatment accelerated Schwann cell differentiation via the estrogen receptors (ERs). **(A)** Immunocytochemistry with anti-MAG (green) displayed the SC differentiation efficacy of the different inducers that were cultured in vehicle or with E2 treatment, or ICI182780 (antagonist of ERs) treatment, or E2 plus ICI182780 treatment. Scale bar, 50 μm. **(B)** Western blotting compared the MAG expression in differentiated cells treated with E2, ICI182780, E2 plus ICI182780 for 1, 2, and 3 days, and vehicle as control. Also shown (Upper) are representative Western blot images, in which β-actin served as an internal standard. ^∗∗^*p* < 0.01 versus vehicle, and ^##^*p* < 0.05 versus E2. One-way ANOVA, *n* = 3 cultures per group. All the data are mean ± SEM.

Proliferation, migration, and differentiation of SCs are important for SC myelination. To examine the effects of E2 on SC proliferation, we added E2 or vehicles (control) to SC culture after 24 h incubation on PDL-coated cover slips or fasciculated DRG axons. SC proliferation was determined by EdU labeling/detection kit. E2 did not change the number of double-labeled cells with EdU and Hoechst, indicating that E2 is not essential for SC proliferation (**Figure 2**, in PDL group, Vehicle: 0.22 ± 0.31 and E2: 0.78 ± 0.63, *p* = 0.8756, Student’s *t*-test; in axonal membrane group, Vehicle: 39.07 ± 9.30 and E2: 40.62 ± 6.67, *p* = 0.9491, Student’s *t*-test). To investigate the effects of E2 on SC migration, we quantified reaggregated Schwann cells on fasciculated DRG axons or on PDL-coated cover slips. As shown in **Figure 3**, E2 had no effects on SC migration at both culture conditions. As a positive control, HRG significantly increased SC migration in PDL group (**Figure 3B**, Vehicle: 1 ± 0.03, E2: 1.01 ± 0.05 and HRG: 1.98 ± 0.12, *p* = 0.0007, One-way ANOVA). However, this effect was not found in DRG axons group (**Figure 3C**, Vehicle: 1 ± 0.12, E2: 0.97 ± 0.04 and HRG: 0.97 ± 0.07, *p* = 0.9317, One-way ANOVA), which may due to DRG neurons can release kinds of cytokines and neurotrophins in the co-culture system and these factors may compensate the role of HRG. To induce SC differentiation, we added 1 mM db-cAMP and 20 ng/ml HRG to SC culture. The results of western blotting and immunocytochemistry showed that the myelin protein MAG is highly enriched in differentiated SCs 3 days after induction (**Supplementary Figure S1**). To characterize the effects of E2 on SCs differentiation, SCs were treated with E2 or ICI182780 (antagonist of ERs), and the expression of MAG was evaluated after stimulation. The results showed that the expression of MAG increased upon E2 treatment, while the expression of MAG was inhibited after treatment with ICI1827 80 (**Figure 4**, Vehicle: 0.97 ± 0.02, E2: 1.09 ± 0.03, E2 + ICI: 1.01 ± 0.08 and ICI: 1.00 ± 0.04 in 24 h, *p* = 0.8718, One-way ANOVA; Vehicle: 0.95 ± 0.03, E2: 1.04 ± 0.07, E2 + ICI: 0.93 ± 0.03 and ICI: 0.90 ± 0.02 in 48 h, *p* = 0.7059, One-way ANOVA; Vehicle: 1.01 ± 0.01, E2: 1.29 ± 0.02, E2 + ICI: 1.00 ± 0.06 and ICI: 0.93 ± 0.08 in 72 h, *p* = 0.0076, One-way ANOVA).

Together, we may therefore conclude that E2 promoted SC differentiation rather than proliferation or migration to induce myelination.

### E2 mediates SC Differentiation by Activating ERβ and Further ERk1/2

**FIGURE 5:**
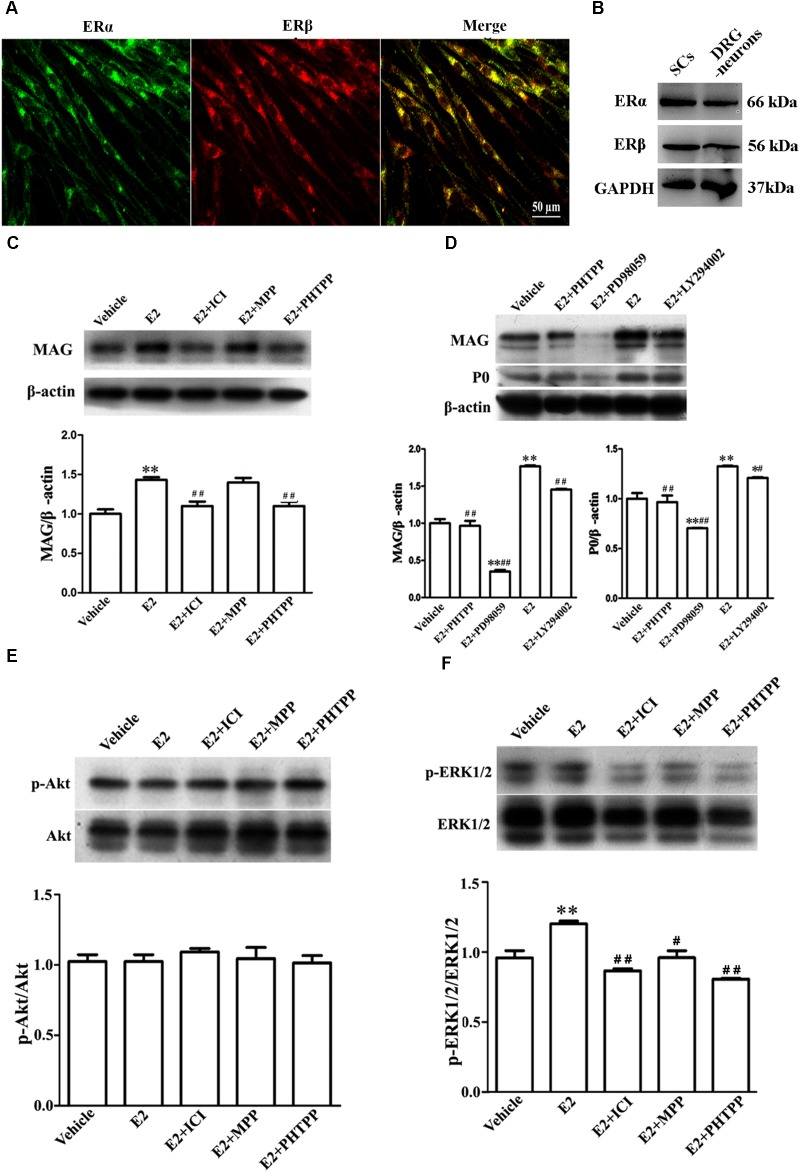
E2 enhances Schwann cell differentiation via ERβ-ERK1/2 signaling. **(A)** Immunohistochemistry with anti-ERα (green) and anti-ERβ (red) of Schwann cells. Scale bar, 50 μm. **(B)** ERα and ERβ expression in the cultured pure Schwann cells and DRG neurons s, as shown by western blotting. **(C)** Western blotting compared the MAG expression in differentiated SCs treated with E2 alone or with E2 plus ICI182780 or MPP (antagonist of ERα) or PHTPP (antagonist of ERβ) for 3 days. **(D)** Western blotting compared the MAG and P0 expression in differentiated SCs treated with E2 alone or with E2 plus PHTPP or PD98059 (antagonist of MEK1/2) or LY294002 (antagonist of PI3K) for 3 days, and vehicle as control, β-actin served as an internal standard. **(E,F)** Showed the phosphorylation of AKT **(E)** and ERK 1/2 **(F)** in differentiated SCs after treatments with E2 alone or with E2 plus ICI182780 or MPP or PHTPP for 3 days. ^∗∗^*p* < 0.01 versus vehicle, and ^#^*p* < 0.05, ^##^*p* < 0.01 versus E2. One-way ANOVA, *n* = 3 cultures per group. All the data are mean ± SEM.

E2 initiates the signaling events through the estrogen receptors (ERs). The expression of ERs in SCs was evaluated by immunohistochemistry and western blotting of the estrogen receptor alpha (ERα) and beta (ERβ). We found that both ERα and ERβ were expressed in the SCs (**Figures 5A,B**).

To characterize which E2R isoform was mainly responsible for SC differentiation, the influences of E2 alone or E2 plus ICI182780 or MPP (ERα antagonist) or PHTPP (ERβ antagonist) on myelin protein MAG expression were analyzed by western blotting. As seen in **Figure 5C**, the protein level of MAG (1 ± 0.05) was increased when cells were treated with E2 alone (1.40 ± 0.04) or E2 plus MPP (1.38 ± 0.06, *p* = 0.0081, One-way ANOVA), whereas MAG upregulation was abolished in the presence of the inhibitor ICI182780 (1.10 ± 0.05) or PHTPP (1.07 ± 0.04, *p* = 0.0053, One-way ANOVA). These results indicated that ERβ not ERα is essential for promoting SC differentiation.

The ERs function by activating internal signaling transduction, for example the cascades of PI3K/AKT and ERK/MAPK (Maggi et al., 2004). Therefore, we analyzed whether E2 could promote SC differentiation via the pathway of PI3K/AKT or ERK/MAPK. The phosphorylation of both ERK1/2 and AKT were detectable already after 5 min incubation with E2 (**Supplementary Figure S2**). We used ERK1/2 inhibitor PD98059 and AKT inhibitor LY294002 to further determine through which signaling pathway E2 regulates SC differentiation. As shown by western blotting, in the presence of PD98059, the MAG protein level was reduced by 82% ± 6.7 (E2: 1.76 ± 0.02, E2 + PD98059: 0.32 ± 0.02, *p* = 0.0047, One-way ANOVA) and the P0 protein level was reduced by 40% ± 4.5 (E2: 1.34 ± 0.01, E2 + PD98059: 0.71 ± 0.01, *p* = 0.009, One-way ANOVA) (**Figure 5D**), suggesting that the phosphorylation of ERK1/2 mediated the effects of E2 on SC differentiation. On the contrary, the AKT inhibitor LY294002 had no effects on E2-induced myelination (**Figure 5D**, MAG: 1.48 ± 0.02; P0: 1.22 ± 0.01). We further determined the phosphorylation levels of ERK1/2 and AKT in differentiated SCs when treated with E2 alone, E2 plus ICI182780, MPP or PHTPP for 3 days. We found that the expression of pERK1/2 rather than pAKT was significantly increased after 3 days E2 treatment and that E2-induced upregulation of pERK1/2 was abolished by the treatment with ICI182780, MPP or PHTPP (**Figure 5E**: Vehicle 1.04 ± 0.03, E2 1.02 ± 0.03, E2 + ICI 1.09 ± 0.02, E2 + MPP 1.05 ± 0.07, E2 + PHTPP 1.01 ± 0.03, *p* = 0.7692, One-way ANOVA; **Figure 5F**: Vehicle 0.97 ± 0.04, E2 1.21 ± 0.02, E2 + ICI 0.87 ± 0.01, E2 + MPP 0.96 ± 0.04, E2 + PHTPP 0.81 ± 0.01, *p* = 0.0053, One-way ANOVA).

Overall, these results demonstrated that E2-induced SC differentiation may be mediated by ERβ and ERK1/2 activation.

### E2 Promotes Remyelination in the Regenerated Sciatic Nerves

**FIGURE 6:**
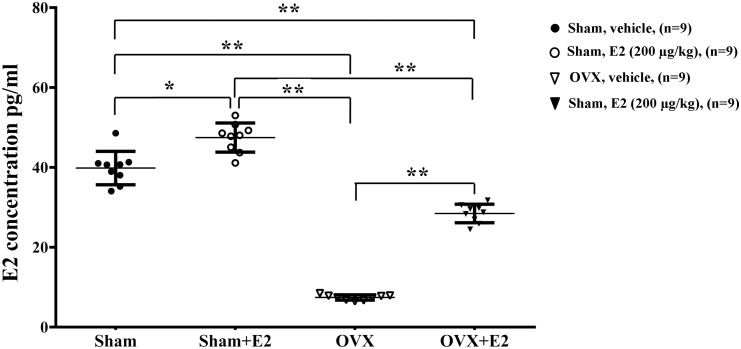
Serum levels of E2 in female rat. Serum was collected 24 h after subcutaneous injection of E2 (0.2 μg/g) or vehicle (saline) in OVX or sham mice, and measurement of E2 in the serum was conducted by ELISA. ^∗^*p* < 0.05, ^∗∗^*p* < 0.01 versus sham. One-way ANOVA, *n* = 5 rats per group. All the data are mean ± SEM.

**FIGURE 7:**
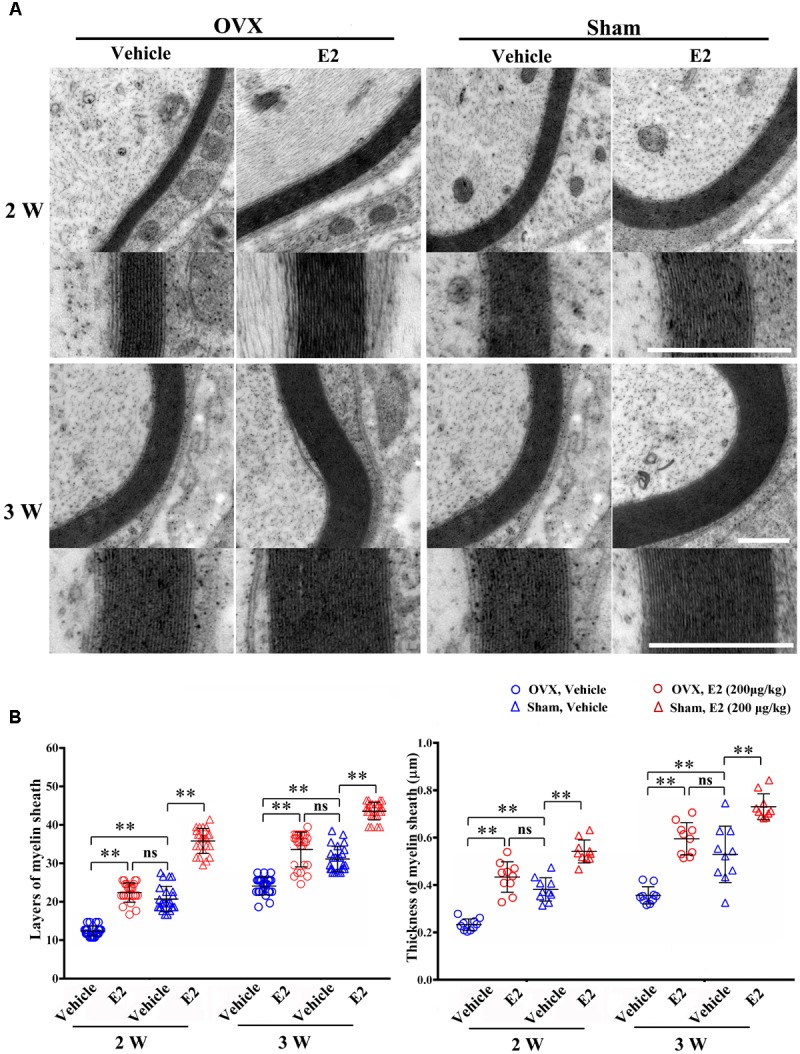
E2 promotes axon remyelination in regeneration sciatic nerve. **(A)** Representative transmission electron micrographs, obtained at 2 and 3 weeks post-surgery, of regenerated nerve in the OVX, OVX treated with E2, sham, sham treated with E2 groups, scale bar, 0.6 μm. **(B)** Histograms comparing the number of myelin sheath layers and the thickness of the myelin sheath among the OVX, OVX treated with E2, sham, sham treated with E2 groups. ^∗∗^*p* < 0.01 versus vehicle. Two-way ANOVA, *n* = 5 rats per group. All the data are mean ± SEM.

To determine the effects of E2 on remyelination during the injured nerve regeneration, we ovariectomized female rats to eliminate endogenous estrogens production prior to nerve surgery. The female rat has a 4–5 days estrous cycle, which includes proestrous, estrous, metestrous, and diestrous (Goldman et al., 2007). Several studies have found that the female animal in different phase of cycle has a different effect on physiological and pathological processes in the nervous system (Hayashi et al., 2005; ter Horst et al., 2012). In this study, we identified the stage of estrous cycle and measured serum estradiol levels at each phase via ELISA. We found that the estradiol level was highest in the proestrous (64.78 ± 3.58 pg/ml) and lowest in the diestrous (14.81 ± 5.15 pg/ml), the levels in the estrus (44.7 ± 6.39 pg/ml) and metestrus (27.01 ± 5.27 pg/ml) was in between. For this paper, we chose the female rats in the estrous to do the ovariectomy. Following the sciatic nerve crush surgery, E2 (0.2 μg/g) or saline was injected subcutaneously every day in OVX and sham groups. To confirm the effectiveness of E2 treatment, we measured the serum levels of E2 by ELISA at 1 day after E2 or vehicle treatment in all four groups. As shown in **Figure 6**, the E2 level in OVX group (7.57 ± 1.12 pg/ml) was significantly lower than that in the Sham group (40.94 ± 5.63 pg/ml. The E2 level in OVX group receiving E2 was significantly increased to 27.56 ± 2.98 pg/ml, and the E2 level in the Sham group receiving E2 was also moderately increased to 49.10 ± 1.56 pg/ml (*p* = 0.0074, One-way ANOVA). To analyze the effects of E2 on myelin regeneration, we used electron microscopy to observe the distribution of the myelin sheaths in these four groups at 2 and 3 weeks after nerve injury. Both the number and thickness of myelin sheaths were significantly increased when animals treated with E2 (**Figures 7A,B**. Layers of myelin sheath at 2w: OVX Vehicle 12.29 ± 1.23, OVX E2 22.35 ± 2.35, Sham Vehicle 21.01 ± 2.68, Sham E2 36.03 ± 2.91, *p* = 0.00009, Two-way ANOVA; Layers of myelin sheath at 3w: OVX Vehicle 24.13 ± 2.23, OVX E2 33.35 ± 4.58, Sham Vehicle 31.34 ± 2.91, Sham E2 43.74 ± 2.40, *p* = 0.00005, Two-way ANOVA. Thickness of myelin sheath at 2w: OVX Vehicle 0.23 ± 0.02, OVX E2 0.43 ± 0.06, Sham Vehicle 0.37 ± 0.05, Sham E2 0.54 ± 0.04, *p* = 0.00011, Two-way ANOVA; Thickness of myelin sheath at 3w: OVX Vehicle 0.35 ± 0.04, OVX E2 0.6 ± 0.07, Sham Vehicle 0.53 ± 0.11, Sham E2 0.74 ± 0.05, *p* = 0.000074, Two-way ANOVA).

### E2 Increases the Number of Lysosomes Containing the Myelin Protein P0

**FIGURE 8:**
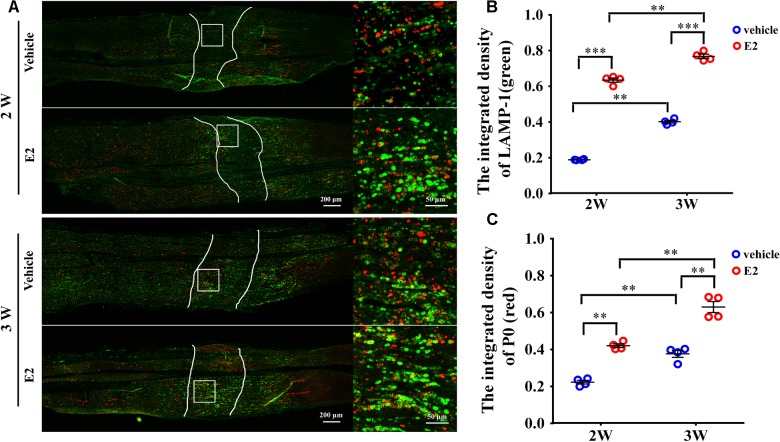
E2 increased the number of lysosomes containing myelin protein P0 in regeneration sciatic nerve. **(A)** Immunohistochemistry with anti-LAMP1 (green) and anti-P0 (red), obtained at 2 and 3 weeks after surgery, of the regenerating nerve in OVX rats with E2 or vehicle treatment. Also shown are the higher magnifications of the boxed areas. It is worth noting that P0 has high colocalization ratio with LAMP1 in E2-treated group. Scale bars, 200 μm; zoom in, 50 μm. **(B,C)** The expression of lysosome membrane protein LAMP1 and myelin protein P0 in the regenerating region of E2-treated group was significantly higher than vehicle group. ^∗∗^*p* < 0.01, ^∗∗∗^*p* < 0.001 versus vehicle. Two-way ANOVA, *n* = 12 slices from 4 rats per group. All the data are mean ± SEM.

Early studies found that the myelin protein P0 was stored in the lysosomes of Schwann cells and can be transported to the plasma membranes via lysosomal exocytosis, which contributes to remyelination during the injured sciatic nerve regeneration (Chen et al., 2012). To determine whether myelin protein trafficking by SC lysosomes could be affected by E2, we analyzed double immunostaining of lysosomal-associated membrane protein 1 (LAMP-1), primarily residing across lysosomal membranes, and the myelin protein P0 at 2 and 3 weeks after nerve injury. In the longitudinal section slices of injured sciatic nerves, sporadic signals of LAMP-1 and P0 were visible in the nerve regenerating region of control groups, whereas LAMP-1 and P0 signals were significantly increased in E2-treated groups at both 2 and 3 weeks after injury (**Figure 8**. The integrated density of Lamp-1 at 2w: Vehicle 0.18 ± 0.01, E2 0.65 ± 0.01; the integrated density of Lamp-1 at 3w: Vehicle 0.41 ± 0.01, E2 0.78 ± 0.01. *p* = 0.0064, Two-way ANOVA. The integrated density of P0 at 2w: Vehicle 0.22 ± 0.01, E2 0.42 ± 0.01; the integrated density of P0 at 3w: Vehicle 0.38 ± 0.01, E2 0.64 ± 0.03, *p* = 0.0097, Two-way ANOVA). Also, we found that P0 has higher degree of colocalization with LAMP1 in the regenerating region of E2-treated animals (**Figure 8**). These results indicated that E2 may accelerate remyelination of the regenerated sciatic nerves by increases the number of lysosomes in Schwann cells, which lysosomes contain myelin protein and transport myelin protein to the cell membrane through exocytosis.

## Discussion

The lipophilic steroid hormone 17β-estradiol (E2) can penetrate the blood–brain barrier. Therefore, its role for neuroprotection against brain damages or degeneration has been extensively studied (Garcia-Segura et al., 2001; Pansiot et al., 2016). E2 also protects the brain from demyelination and stimulates remyelination by acting on oligodendrocytes, which may explain why women have better prognosis than men in multiple sclerosis (Acs et al., 2009; Hirahara et al., 2009; Taylor et al., 2010; Kipp et al., 2012; Kumar et al., 2013; Patel et al., 2013). However, little is known about the role of E2 in Schwann cells myelination and remyelination in the PNS (Zhu and Glaser, 2008; Chen et al., 2016). The present work found that E2 not only promotes SC myelination in a coculture model of SCs/DRG neurons, but also promotes SC remyelination in a rat sciatic nerve crush model.

Co-cultures systems with DRG explants/Schwann cells are widely used to study myelination *in vitro*. Using this well-designed model, we found that the number of myelins and the expression levels of MAG were increased after 21 days treatment with E2. This indicates that E2 promotes myelin formation. To explore the mechanisms of actions of E2, we further characterize the changes in SC proliferation, migration, and differentiation. Our results indicated that E2 only promotes SC differentiation but not proliferation and migration, which is not consistent with previous reports that E2 promotes SC proliferation and differentiation (Chen et al., 2016). The reason is still not clear and maybe due to the different culture conditions.

One of the interesting findings in this article is that E2 can promote SC differentiation through the ERβ-ERK1/2 signaling pathway. E2 exerts it biological function by binding to the estrogen receptors. ERα and ERβ, as the classic ERs, can mediate both genomic and non-genomic effects of estrogen. Previous studies demonstrated that activation of ERβ may improve CNS remyelination by enhancing oligodendrocyte differentiation (Crawford et al., 2010). Enrichment of ERα and ERβ in primary SCs, as shown by immunohistochemistry, is consistent with previous findings (Fujimoto et al., 2004). In the present study, we used selective ER agonist to further identify which specific ER mediated the effects of E2 on myelination in SCs. We found that the effects of E2 on promoting SC differentiation was abolished by ERs antagonist ICI182780 or ERβ antagonist PHTPP but not by ERα antagonist MPP. Although pERK1/2 and pAKT were both detectable within 5 min after addition of E2, only pERK1/2 but not pAKT expression was significantly increased after 3 days E2 treatment. E2-induced upregulation of myelin protein MAG and P0 were abolished by the treatment with ERK1/2 inhibitor PD98059 not by the AKT inhibitor LY294002. Furthermore, E2-induced upregulation of pERK1/2 was abolished by the treatment with ICI182780 and PHTPP. These suggested that the ERβ-ERK1/2 signaling pathway plays a key role in E2-induced SC differentiation. Interestingly, a previous report found that selective ERβ agonists activated the PI3K/AKT/m TOR signaling pathway in oligodendrocyte differentiation and promoted remyelination in the CNS of mice with multiple sclerosis (Kumar et al., 2013). These results suggest that the mechanisms by which E2 promotes myelination in oligodendrocytes and Schwann cells may be different.

An increase in transportation of myelin proteins to the plasma membranes may be essential for E2-induced remyelination, which is dependent on the E2-induced increase in the number of lysosomes. Lysosome of oligodendrocytes and Schwann cells contain abundant myelin proteins, such as PLP and P0, playing an important role in the remyelination of injured nerve via lysosomal exocytosis (Chen et al., 2012; Shen et al., 2016; Su et al., 2016). We also found that the expression of these two lysosome markers LAMP-1 and myelin protein P0 were significantly increased in E2-treated rats compared with those in vehicle-treated rats after sciatic nerve crush injury. In addition, P0 is highly colocalized with LAMP1 in the regenerating region of E2-treated group. The mechanism by which E2 regulates myelin protein trafficking and lysosomal exocytosis could be an interesting topic to explore for future research. Finally, E2 administration promotes remyelination in the sciatic nerve crush model in both OVX and non-OVX female rats, which indicates that exogenous E2 can augment myelin formation.

Overall, our results suggest that E2 administration may be a novel therapeutic approach for remyelination enhancement after nerve injury.

## Author Contributions

YG and GC designed the experiments, drafted the article, and approved the final version to be submitted. YG, GC, and LX drafted the article. YW conducted most of experiments including cell culture, western blotting, rat breeding, and behavioral test. WS established the *in vitro* myelination model. YS, XH, LL, XT, and YY did other experiments and analyzed the data.

## Conflict of Interest Statement

The authors declare that the research was conducted in the absence of any commercial or financial relationships that could be construed as a potential conflict of interest.
