# Supplementary material for: 17β-Estradiol Enhances Schwann Cell Differentiation via the ERβ-ERK1/2 Signaling Pathway and Promotes Remyelination in Injured Sciatic Nerves
Source: Front Pharmacol. 2018 Oct 9;9:1026. doi: 10.3389/fphar.2018.01026 (PMC6189327; doi:10.3389/fphar.2018.01026)
Supplement: Supplementary file 1 [file Table_1.DOCX]

**17β-Estradiol enhances Schwann cell differentiation via the ERβ-ERK1/2 signaling pathway and promotes remyelination in injured sciatic nerves**

Yun Gu, Yumen Wu, Wenfeng Su, LingYan Xing, Yuntian Shen, Xiaowen He, Ying Yuan, Xin Tang, Gang Chen

Supplementary Figures (1-2)

**Fig S1**


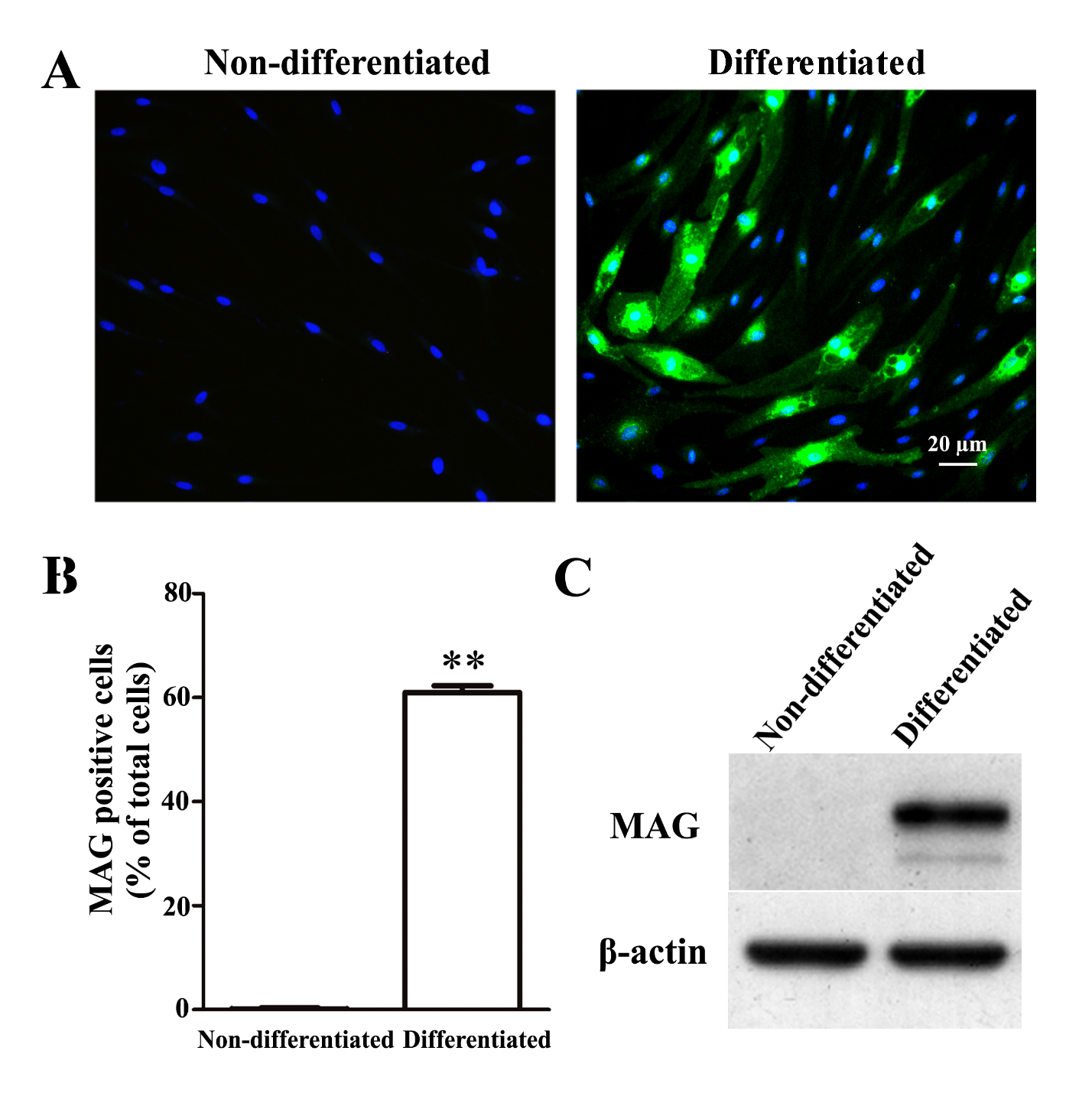


**Supplementary Figure 1. The myelin protein MAG is highly enriched in differentiated SCs 3 days after induction.** (A) Double staining showing increased MAG (green) expression in differentiated SCs 3 days after induction. Scale, 20 μm. (B) Number of MAG-positive SCs in differentiated SCs 3 days after induction. **p<0.01, Student’s t-test, n = 3 cultures per group. All the data are mean ± s.e.m. (C) Western blotting shows MAG upregulation in differentiated SCs 3 days after induction.

**Fig S2**

**
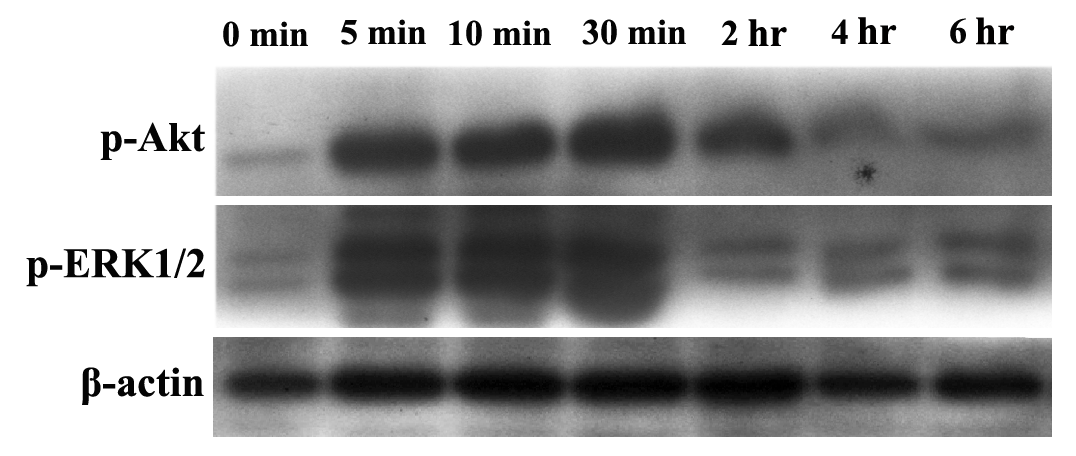
**

**Supplementary Figure 2. Western blotting shows the phosphorylation of ERK1/2 and AKT upregulation in cultured SCs within 5 mins after E2 treatment.**
